# Dunn’s Model of Sensory Processing: An Investigation of the Axes of the Four-Quadrant Model in Healthy Adults

**DOI:** 10.3390/brainsci9020035

**Published:** 2019-02-07

**Authors:** Alexia E. Metz, Daniella Boling, Ashley DeVore, Holly Holladay, Jo Fu Liao, Karen Vander Vlutch

**Affiliations:** Occupational Therapy Doctoral Program, School of Exercise & Rehabilitation Sciences, College of Health & Human Services, University of Toledo, Toledo, OH 43606, USA; daniella.boling@rockets.utoledo.edu (D.B.); ashley.devore@utoledo.edu (A.D.); holly.holladay@rockets.utoledo.edu (H.H.); jofu.liao@rockets.utoledo.edu (J.F.L.); karen.karmol@rockets.utoledo.edu (K.V.V.)

**Keywords:** sensory processing

## Abstract

We examined the behavioral response (BR) and threshold (T) axes of Dunn’s four-quadrant model of sensory processing (1997). We assessed whether they are ordinal ranges and if variation is associated with other similarly described characteristics: Introversion/Extraversion (I/E) of Eysenck’s personality model (Sato, 2005), and somatosensory event related potentials (SERP) and their gating (Davies & Gavin, 2007). From healthy adults (*n* = 139), we obtained: Adult/Adolescent Profile (A/ASP, Brown & Dunn, 2002) and Eysenck’s Personality Questionnaire, Brief Version (Sato, 2005) scores and peak amplitude and gating factor of SERP P50. We found that BR scores did not differ across normative categories of the A/ASP, but T scores significantly increased along the axis. I/E scores did not vary with BR scores. There were no differences or correlations in P50 amplitudes and gating with T scores. The findings suggest that the BR axis may not reflect a construct with ordinal range, but the T axis may. Dunn’s concept of BR appears to be distinct from Eysenck’s concept of I/E. SERP and its gating may not be directly reflective of sensory processing thresholds in healthy adults. Conclusions are limited by having few participants with passive behavior regulation or low threshold patterns of processing.

## 1. Introduction

Occupational therapist Dr. Jean A. Ayres was one of the first to bring attention to sensory processing’s role in everyday function. Ayres described sensory integration as a behavioral response to sensory input and “explored the association between sensory processing and the behavior of children with learning, developmental, emotional, and other disabilities” [1] (p. 136). Since Ayres’ theory in 1963, there have been many others who have further developed her theory to make advancements in the understanding of sensory processing. Occupational therapist Winnie Dunn created one of the most recognized models of sensory processing called Dunn’s Four Quadrant Model of Sensory Processing. Dunn’s Four Quadrant Model of Sensory Processing is based on two constructs: neurological thresholds and behavioral response. The neurological threshold construct refers to the threshold for response to a sensory stimulus and is described as ranging on a continuum from low to high. Individuals with low sensory thresholds may be quick to notice and respond to stimuli because their systems are easily activated by sensory stimuli. Individuals with high thresholds may not be as responsive, so they may miss stimuli that others respond to. Dunn suggests that neural regulation occurs as a balance of excitation and inhibition which creates thresholds for response. Each person has unique thresholds for responding to sensory information. Thresholds may not be the same for all sensory modalities. The second construct, behavioral response, also exists on a continuum based upon whether people have passive or active strategies in response to their environments. Though individuals with passive tendencies may internally respond to stimuli, they might not take action to change their environments, whereas at the active end of the continuum, individuals may tend to actively control the type and amount of sensory input in their environments [2]. Dunn theorized that these constructs may be influenced by both genetic and environmental factors [2].

These two constructs serve as axes that cross to form quadrants making Dunn’s Four Quadrant Model of Sensory Processing [3]. The top, left quadrant arises from a combination of high neural thresholds and passive responding strategies. This is called low registration. The top, right quadrant is called Sensory Seeking with a pattern of high thresholds and active responding strategies. The bottom, left quadrant is the sensory sensitivity category with low thresholds and passive responding strategies. The bottom, right quadrant is a pattern with low thresholds and active responding strategies, referred to as sensory avoiding [1,2,3,4,5].

Disruption in an individual’s sensory processing ability can affect engagement in occupations of daily life [6,7,8]. As Brown and Dunn state, “areas of concern emerge only when a person’s sensory processing pattern seems incompatible with his or her desired or necessary life activity choices” [7] (p. 5). This is when sensory processing becomes a concern of occupational therapy. According to the Sensory Processing Disorder Foundation [9], the term Sensory Processing Disorder (SPD) is used to describe a condition in which a person encounters problems with his/her daily occupations due to how he/ she processes and responds to sensory information. The American Psychological Association’s review committee for the Diagnostic and Statistical Manual of Mental Disorders did not include SPD in the fifth edition because they judged that there was insufficient evidence supporting it as a stand-alone disorder [10,11]. Currently SPD has been recognized as a distinct disorder by Diagnostic Classification: Zero to Three: Diagnostic Classification of Mental Health in Developmental Disorders in Infancy and Early Childhood [12] and the Diagnostic Manual for Infancy and Early Childhood [10,13]. Standardized assessments of sensory processing, such as Dunn’s Sensory Profile [14], demonstrate normal distribution in scores. This allows clinicians to interpret scores falling outside of the first and second standard deviation as likely and probable indicators of SPD. Measured this way, SPD has been found to be present in a conservative estimate of 5% of the general population [15] and is associated with a variety of diagnoses, including attention deficit hyperactivity disorder, Fragile X syndrome, autism spectrum disorders, developmental disability [16], dysfunctional elimination syndrome [17], and major affective disorders such as depression [18,19].

Dunn and Brown [2,14] conducted studies to compare children with and without disabilities according to Dunn’s model. Using factor analysis, they found that some typically developing children had similar sensory modulation patterns that could be observed in children with various disabilities. Similarly, Kinnealy, Koenig, and Smith [20] found that healthy adults with Sensory Profile scores indicative of low thresholds reported lower quality of life indicators on the Short Form-36 Health Survey [21]. Together, this provides evidence that intense sensory processing patterns are not exclusive to people diagnosed with disabilities. The focus of intervention should not be on the disability, or lack thereof, but instead on how the person functions in his/her environment given his/her intense sensory processing patterns [2]. Engel-Yeger, Serafini, and colleagues [18,19] found patterns of association between distinct characteristics of major affective disorders (such as depression, anxiety, irritability, impulsivity, alexithymia, and hopelessness) with scores indicative of extreme sensory processing patterns with Dunn’s model. They suggest that sensory processing patterns may be trait markers, which allows tailoring of intervention.

As part of a study of the construct validity of the Adult/Adolescent Sensory Profile, Brown, and colleagues [3] used the physiological measure of skin conductance to examine response and habituation to stimuli as a proxy for the construct of neurological threshold. The researchers delivered a repeated auditory stimulus to participants while measuring skin conductance. Results indicated that people with sensory sensitivity and sensory avoiding patterns were more responsive, as measured by changes in skin conductance, than people with low registration and sensory seeking patterns. They also found that people with sensory sensitivity and sensory seeking patterns took more trials to habituate to the stimuli than people with the sensation avoiding and low registration patterns [3]. As the Dunn’s model is widely used in clinical settings for assessment, client education, and intervention planning, it is important that its underlying constructs are tested. One way to validate a model is to compare its constructs to external measurements that are similarly described.

Dunn’s behavior response axis describes individuals’ characteristic actions taken in response to sensory stimulation. The language in which it is described is reminiscent of the personality characteristic of introversion/extraversion, which reflects how individuals’ respond to social stimulation. Psychologist Eysenck [21,22] developed a four-quadrant personality model, which described introversion/extroversion on a horizontal axis and neuroticism on a vertical axis [23]. He described individuals who seek stimulation due to a low arousal level as extraverts, and individuals who avoid stimulation due to naturally high arousal levels as introverts [24]. Neuroticism was viewed on a continuum from low neuroticism describing individuals whose emotions fluctuate very little and high neuroticism describing individuals whose emotions fluctuate greatly [24]. Sato [23] created the Eysenck Personality Questionnaire-Brief Version based on the Eysenck Personality Questionnaire-Short Version, one of several versions based on Eysenck’s personality theories and original personality questionnaire. The EPQ-BV measures the two main personality traits from Eysenck’s theory: introversion/extraversion and neuroticism [24].

The similarities in the verbiage of Dunn’s behavioral response aspect of sensory processing and the introversion/extraversion aspects of Eysenck’s personality model warrant exploration of whether the latter can be used to validate the former. Sato, in his revision of the Eysenck Personality Questionnaire to create a brief version, summarized the Eysenck’s research on the personality dimensions of introversion and extroversion: 

“Compared to introverted individuals, extraverted individuals naturally have a lower arousal level. This makes extraverted individuals seek stimulation to raise their arousal level. In contrast, the naturally high arousal level of introverted individuals makes them avoid stimulation as much as possible. This is considered to be why introverts tend to like quiet activities while extraverts tend to like stimulating activities” [24] (p. 106)

What Sato termed “*arousal level*” in his summary, Eysenck [25] may be interpreted as referring to “*sensory threshold*” (p. 100). Dunn describes sensory processing differences similarly to Eysenck’s ideas on introversion and extraversion:

“Young children with high ‘sensory’ thresholds would respond to very few stimuli, while young children with low thresholds would respond to many stimuli. On the other end of the continuum, young children can respond to counteract their thresholds, these children might either try to exert excessive energy seeking stimuli to try to meet high thresholds or exert energy to avoid triggering low thresholds.” [4] (p. 27)

Both authors refer to sensory thresholds and the way in which individuals respond to sensory information in similar manners; leading to the possibility that what Eysenck refers to as the continuum between introversion and extraversion may be the same construct as what Dunn refers to as behavioral regulation based on sensory threshold.

The description of the threshold axis invokes neurological processing of sensory stimuli. Physiological measurements of sensory responses may be useful in assessing this axis. Sensory gating, a process related to modulation, is associated with functions of attention, processing speed, and working memory [26]. This is a neural process by which the brain responds to stimuli and filters out extraneous information. This is often measured by encephalography studies. Electroencephalography (EEG) records electrical brain activity through electrodes placed on the scalp. Event related potentials (ERPs) are obtained by giving a sensory stimulus multiple times and averaging the EEG segments surrounding the event [27]. Changes in the magnitude of ERPs in response to repeated stimuli are indicative of the brain’s modulation of sensory stimulation, called gating. Many studies use an ERP called the P50 as their measure of sensory gating. The P50 is a positive going wave which generally occurs in the cortex 40–90 milliseconds after the stimulus. It is thought that the amplitude of P50 may reflect the degree of neural processing afforded to the stimulus. The amount of sensory gating that occurs for a pair of stimuli is represented as a ratio that is measured by dividing the peak amplitude of the P50 of the second stimulus as a proportion of the peak amplitude of the P50 of the first stimulus. The first stimulus is generally referred to as ‘*conditioning’* and the second stimulus is the ‘*test’*, thus the ratio is called a T/C ratio. A smaller T/C ratio is indicative that sensory gating has occurred [26].

Davies and Gavin used EEG to test the diagnostic validity of sensory processing disorder. In 2007, Davies and Gavin tested the hypothesis that behavioral dysfunction in children with sensory processing disorders is related to dysfunction in brain function. To do this, they used the P50 gating paradigm with children with SPD, as verified by Dunn’s Sensory Profile, and typically developing children. Children with SPD showed less sensory gating (mean T/C ratio of 0.77 ± 0.42) than typically developed children (mean 0.58 ± 0.31, *p* < 0.05). This suggests that children with SPD may have less ability to filter out extraneous sensory input. In addition, children with SPD were either hyper-responsive or hypo-responsive to the stimulus. In a cross-sectional analysis, the researchers factored in maturation as a variable in sensory processing. Results indicated that gating was not improved in older children with SPD as it was in older typically developing children [28]. A subsequent study by Davies, Chang, and Gavin [26] reports that children with SPD showed disorganized patterns of brain activity in response to both intensity and frequency of stimuli compared to typically developed peers.

Dunn’s model has clinical utility, and the associated standardized assessment has strong psychometrics. In this study, we examined the constructs that underlie the model, behavioral response and threshold. We tested the hypotheses that these axes of the model, as measured by the sensory profile, are ordinal ranges and that variation along the ranges is consistent with variation in external measures of characteristics that may also reflect sensory processing as described by Dunn. To begin, we converted raw scores of the Adult/Adolescent sensory profile to axis scores. To assess whether axis scores were consistent with the clinical presentation of Sensory Profile results, we compared the derived scores and quadrants to the raw and normative scores of the sensory profile. Next, we compared variation in behavioral response and threshold with variation in introversion/extraversion axis of Eysenck’s personality model [23], respectively. Finally, we compared variation in threshold to variation in somatosensory event related potentials and sensory gating.

## 2. Methods

This study was approved by the Institutional Review Board of the University of Toledo, adhering to the Helsinki Declaration (BME-IRB Protocol #107533). The study was conducted in a campus laboratory. We employed a cross sectional research design. Data collected included descriptive demographics, measurements for inclusion/exclusion, standardized questionnaire responses, and somatosensory event related potentials.

### 2.1. Participants

Participants were a recruited convenience sample of adults. Recruiting occurred via word of mouth and flyer, generally focused on greater Toledo, Ohio area. Inclusion criteria were adult age (18–65 years); non-smoking; healthy (no past or current neurological diagnoses nor current major medical diagnoses); intact cognition (by scoring at least 25 on the Mini Mental State Examination (MMSE) [29]; near visual acuity of at least 40/20, with correction as needed, at 14”; expressed hand dominance (left or right); and disclosure of regular medications. Exclusion criteria were being of minor or senior status, regular smoking, scoring lower than 25 on the MMSE, visual acuity poorer than 40/20 even with correction, ambidextrous (by report), or unwillingness to disclose medications. All criterial were screened following informed consent and before any data collection procedures began. Participants who were excused, having not met inclusion criteria, and who completed the study were given a $10 gift card to a coffee retail chain.

### 2.2. Procedure

Following informed consent and inclusion screening, data were collected in two visits, conducted in counterbalanced random order. In one visit, participants completed the Adult/Adolescent Sensory Profile (A/ASP) [7] and the Eysenck Personality Questionnaire, Brief Version (EPQ-BV) [23] via computer-based administration. In the other visit, electroencephalography (EEG) and median nerve stimulation (MNS) were used to collect somatosensory event related potentials (SERP) and sensory gating data. In each session, elements were ordered in a counterbalanced random manner.

#### 2.2.1. Computer-Based Administration of Standardized Questionnaires

Using E-Prime (Pittsburg, PA, USA), the A/ASP and EPQ-BV were administered via computer and a five-button response pad. Participants were trained for 100% accuracy in using the response pad. Test items were presented individually on a computer monitor, and participants used a response box with five lighted buttons to enter their responses. As they entered responses, participants were presented with a review of their responses, item-by-item, and given the opportunity to change their responses until they were satisfied with their response. Final responses were recorded by E-Prime.

#### 2.2.2. Adult/Adolescent Sensory Profile

This assessment includes 60 items, each describing a reaction to sensory stimulation. Respondents use a Likert rating scale ranging from 1, Never (<5% of opportunities), to 5, Always (>95% of opportunities) to describe the frequency of opportunities for which they demonstrate the behavior described by the items. For each quadrant of Dunn’s model, there are 15 items. Total scores in each quadrant can be compared to normative values and reported as within one standard deviation from the mean (similar to others), between one and two standard deviations from the mean (less or more than others), or greater than two standard deviations from the mean (much less or much more than others). Psychometrics for the Sensory Profile include tests for internal consistency, discriminative and convergent validity, and a review by panel of experts [2]. Internal consistency coefficient alphas range from 0.639 to 0.775 for quadrant scores. Discriminative validity was shown through correlations with scores on the New York Longitudinal Study: Adult Temperament Questionnaire [30]. All items on the test were reviewed by an expert panel of five judges to determine if they could be categorized into the four-quadrant model of the original sensory profile. After revision of one item, all items were categorized correctly.

#### 2.2.3. Eysenck Personality Questionnaire, Brief Version

This assessment includes 24 questions about personality traits to which respondents reply on a Likert Scale ranging from 1, ‘not at all’; to 5, extremely. Psychometrics for the EPQ-BV includes correlations with the Eysenck Personality Questionnaire Revised-Short (EPQR-S) version, coefficient alphas, and test-retest reliability values (Sato, 2007). The EPQ-BV shows high correlations with the EPQR-S for the introversion-extraversion scales (Sato, 2007). “The coefficient alphas for the introversion-extraversion and neuroticism scales in the EPQ-BV were 0.93 and 0.91 respectively” (Sato, 2007, p. 115). Finally, the test-retest reliability values for the two scales of the EPQ-BV are 0.93 for introversion-extraversion and 0.91 for the neuroticism scale (Sato, 2007).

#### 2.2.4. Electroencephalography and Median Nerve Stimulation 

Participants refrained from caffeine and social smoking for eight hours prior to EEG sessions. EEG data were recorded via a 24-channel ActiveTwo biopotential measurement system (BioSemi, Amsterdam, The Netherlands). It interfaced with a laptop computer via USB2.0 for data storage and display via ActiView software (Source Signal Imaging, San Diego, CA). Data were sampled at 2 KHz, band pass filtered at 10–200 Hz, and digitized at 24 bits. Using the 10–20 measurement system, 16 well-type scalp electrodes were positioned using a skull cap (Fp1, Fp3, C1, Cz, C3, T7, T1, Tz, T3, T8, P1, Pz, P3, O1, Oz, O3). Surface electrodes were placed at the bilateral mastoids, bilateral temples, Fpz, cervical spine, and bilateral Erb’s points. Water soluble electrolyte gel (Parker Signa, Fairfield, NJ, USA) was used for both types of electrodes.

To obtain stereotyped EEG responses to input to the somatosensory system, we used electrical stimulation of the median nerve at participants’ non-dominate wrist from an Isolated Stimulator (Model DS3, Digitimer, Hertfordshire, England), with contact established via electrolyte gel. Stimulus intensity set at 200% of participants’ detection threshold, established via a stairstep method, provided the stimulation did not evoke movement (thumb twitch) and was tolerated by the participant. In these cases, stimulation as reduced to 175 or 150% of detection. This provided a robust stimulus of the somatosensory system without activating pain or motor fibers of the median nerve. During experiments, MNS was triggered by TTL pulse from the computer running E-Prime software. To distinguish the brain’s response to stimuli from background brain activity and electrical noise, the stimulus must be delivered repeatedly and separate trials are averaged. The occurrences of MNS stimuli were marked in the EEG recordings.

#### 2.2.5. Somatosensory Event Related Potentials

While participants viewed a silent, amusing video, 200 single MNS stimuli were delivered at 5 Hz (200 µs stimuli, delivered at 200 ms start-to-start intervals). The duration of stimulus delivery was 60 s, beginning 10 s into the 86-s video.

#### 2.2.6. Sensory Gating

Sensory gating is reduction of the neurological response to a repeated stimulus. While participants viewed a fixation cross, we delivered 200 pairs of MNS. Each stimulus was 200 µs in duration and the second stimulus in each pair began 500 ms after the start of the first. Pairs were delivered in sets of four while participants were presented with a visual fixation cross for 35 s, with pairs beginning two seconds in. Subsequent pairs eight seconds apart (start-to-start). Participants advanced from set to set by pressing any button on the response pad, allowing them to pace themselves and take breaks between sets of four pairs.

### 2.3. Measurement

#### 2.3.1. A/ASP Score Categorization

The sum of scores for the 15 A/ASP items associated with each quadrant were compared to the normative data published with the assessment to be categorized as ‘similar to most people’ (within one standard deviation of the mean), ‘less than most people’ or ‘more than most people’ (between one and two standard deviations below or above the mean, respectively), or ‘much less than most people’ or ‘much more than most people’ (more than 2 standard deviations below or above the mean, respectively).

#### 2.3.2. A/ASP Axis Scores

We converted raw scores of the A/ASP to axis scores as follows: to relate to one quadrant of the model, each item of the sensory profile is indicative of either a high threshold or a low threshold and either a passive or an active behavioral pattern. Accordingly, there are 30 items indicative of a passive behavioral response (15 each from the low registration and sensory sensitivity quadrants) and 30 indicative of a active behavioral response (15 each from sensory avoiding and sensory seeking). Similarly, there are 30 items indicative of low threshold (sensory sensitivity and sensory avoiding) and 30 indicative of high threshold (low registration and sensory seeking). Each item is rated on a 1–5 point scale. To prevent a gap in the distribution, we subtracted one point from each response to create a 0–4 scale. The sums of passive behavioral response and low threshold scores were multiplied by negative one, producing negative scores. The scores of passive and active behavioral response were totaled to derive a behavioral response axis score. The scores for high threshold and low threshold were added together to derive a threshold axis score. Axis scores could range from −120 to 120.

#### 2.3.3. Derived Quadrants 

Participants’ derived quadrants were obtained by plotting their behavioral response and threshold axes scores against one another, where two positive scores would yield a derived quadrant of sensory seeking, negative behavioral response and positive threshold scores would yield a derived quadrant of low registration, two negative scores would yield a derived quadrant of sensory sensitivity, and active behavioral response and negative threshold scores would yield a derived quadrant of sensory avoiding.

#### 2.3.4. EPQ-BV Axis Scores

Of the 24 questions on the EPQ-BV, two were reverse scored according to the author’s instructions (Sato, 2005). Sato’s factor analysis demonstrated that 12 of the items’ weight on the scale for introversion/extraversion and the remaining 12 items relate to the neuroticism scale. Maximum scores on each scale are 60. To prevent a gap in the distribution, we subtracted one point from each response to create a 04 scale, giving a maximum score of 48. To reflect the axis-crossing of Eysenck’s four-quadrant model, range was shifted to −24 to 24.

#### 2.3.5. EEG Data Analysis

EEG data were analyzed using EMSE software (Cortech Solutions, Wilmington, NC, USA). Digitized EEG recordings were referenced to averaged signals from the mastoid electrodes and subsequently to the average of all channels. A polynomial detrend algorithm was used to remove the effects of drift from the referenced. Recordings were bandpass filtered from 10–300 Hz. Artifacts (pulse, eye movement, and blinks) were removed using a template-based routine available in the software package. Recordings were divided into segments time-locked to the delivery of the MNS stimulus (from 100 ms before through 200 ms after the stimulus). Segments were averaged to obtain somatosensory event related potentials (ERP). At the Cz recording site, we selected the P50 waveform for analysis, measuring the peak amplitude of the ERP between 40 and 85 ms after the stimulus. P50 amplitude must be at least 0.5 µV to be included in analysis. For a measure of sensory gating, we calculated the ratio of the amplitude of the P50 evoked by the second stimulus in pairs to that of the wave evoked by the first stimulus in the pair.

### 2.4. Data Analysis

Statistical significance was determined at the α = 0.05 level, and we report effect sizes for significant findings. To assess whether the axis scores we calculated were consistent with the clinical presentation of sensory profile results, we compared the agreement of derived scores and quadrants to the raw scores of the sensory profile using Fisher’s exact tests and chi-squared analysis. We assessed ordinal variation of calculated scores using one-way ANOVA, with effect size reported as ω and post-hoc Tukey and with *t*-test with effect size *r*. We compared variation in behavioral response and threshold with variation in introversion/extraversion and neuroticism of axes the EPQ-BV using *t*-test and Pearson’s correlation. We compared variation in threshold to variation in somatosensory event related potentials and sensory gating using *t*-test and Pearson’s correlation.

## 3. Results

### 3.1. Participant Characteristics

Through our recruiting efforts, 192 individuals expressed interest in participating in the study. Of those, 147 scheduled participation and 142 provided informed consent. One consenting participant did not meet the inclusion criteria, one withdrew because of scheduling difficulties, and one completed participation but withdrew data from analysis. The data from the remaining 139 participants are included in analysis; however, one did not attend the EEG session. See Table 1 for demographic information of the included participants.

### 3.2. Conversion of A/ASP Scores to Axis Scores

We investigated whether the behavioral response and threshold axes of Dunn’s Four-Quadrant Model of sensory processing, as measured by the sensory profile, are ordinal ranges. To begin, we converted raw scores of the Adult/Adolescent Sensory Profile to axis scores for behavioral response and threshold. We assessed whether calculated axis scores reflect the clinical impression the results of the sensory profile provide by comparing these derived scores to participants’ raw scores on the A/ASP. According to the guidelines of the A/ASP, item scores were totaled for 15 test items for each quadrant, and participants were categorized according to the quadrant in which they had the highest raw score. One participant had the same highest score in two quadrants and was therefore not included in analyses. There were strong alignments between the categorization of behavioral response (passive or active) and threshold (low or high) associated with the quadrant in which the participants had the highest raw score and the respective calculated axis score (*p* < 0.001, Fisher’s exact test for both axes, Cramer’s V = 0.608 for behavioral response and 0.555 for threshold, *n* = 138). For each participant, we plotted the calculated behavioral response axis score against the calculated threshold score to derive a quadrant. We measured the degree of alignment between the quadrant where participants had the highest raw score and the derived quadrants using chi-square analysis. The alignment was significant (χ^2^(6) = 72.650, *p* < 0.001, Cramer’s V = 0.513). 

### 3.3. Assessment of Axes as Ordinal Ranges

Having confirmed that the calculated scores and derived quadrants adequately reflect the clinical presentation given by the A/ASP, we assessed whether calculated scores along each axis would demonstrate ordinal scaling (where participants whose scores fell above or below the mean when compared to the published normative data the A/ASP of would have significantly higher or lower calculated axis scores). According to the quadrant with the highest raw score on the A/ASP, participants were categorized by Passive and Active behavioral response. Within these categories, we compared the calculated response axes scores for participants whose raw scores fell into the less than others, similar to others, more than others, and much more than others classifications outlined by the Sensory Profile. For each category (passive and active), there was no difference in the means, as demonstrated with ANOVA (see Table 2). This suggests that calculated behavioral response axis scores may not reflect an ordinal measurement. Therefore, for further analysis relating to the behavioral response axis, participants were grouped according to the quadrant in which they had their highest raw score on the A/ASP.

According to the quadrant with the highest raw score, participants were categorized by Low and High threshold on the A/ASP. Within these categories, we averaged the calculated response axes scores for participants whose raw scores fell into the Less Than Others, Similar to Others, More Than Others, and Much More Than Others classifications outlined by the A/ASP. For the High threshold category, there was a significant difference in calculated threshold axis scores. In post hoc analysis with Tukey’s, scores for participants in the Much More than Others group were significantly higher than scores for those in the Similar to Others group (*p* = 0.017). For the Low threshold category, there was no difference in the means. See Table 3. We compared the calculated axis scores for participants in the Much More than Others High threshold category to all of the participants in the Low threshold group using *t*-test. There was a significant difference (*t*(22) = 8.115, *p* < 0.001, *r* = 0.34). Together, this suggests that the calculated threshold axis score may reflect an ordinal measurement. The distribution of calculated threshold axis scores was normal (skew = −0.041, kurtosis = 0.503). Subsequently, for analyses involving the threshold axis, these data were treated as ordinal values.

**Figure 1:**
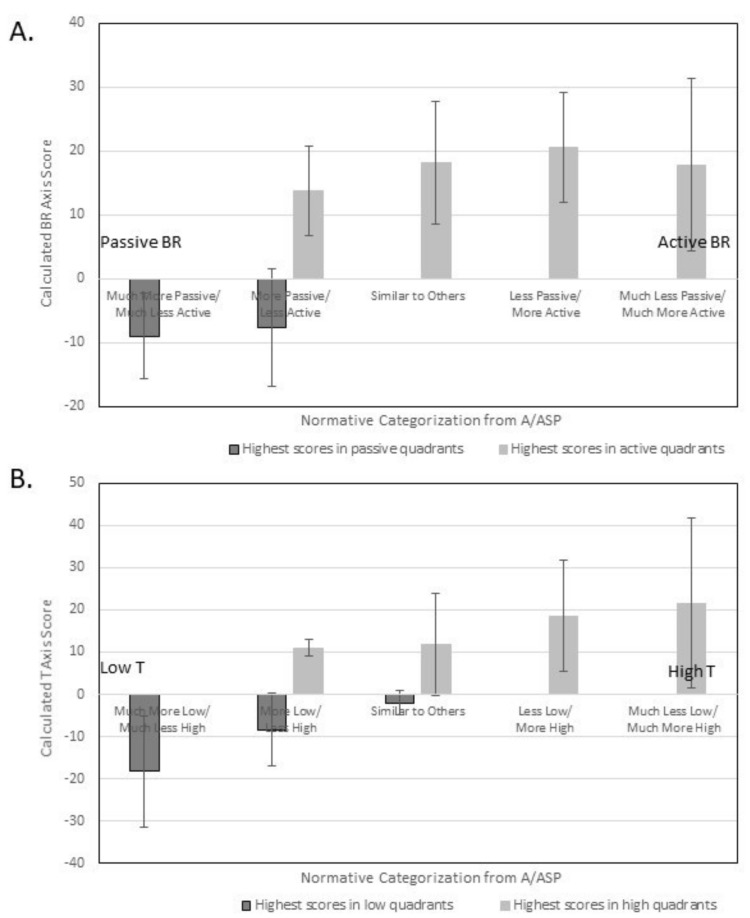
Calculated axis scores for participants according to the quadrant in which they had their highest score on the A/ASP. (**A**) Given the quadrant in which participants had their highest score on the A/ASP, participants were categorized into Passive BR and Active BR, then further divided according to how their score compared to the normative distribution. This bar chart shows the mean and deviation of calculated BR axis scores, arranged along the passive to active spectrum of the axis. The means do not increase from passive to active in an ordinal fashion. (**B**) Given the quadrant in which participants had their highest score on the A/ASP, participants were categorized into Low T and High T, then further divided according to how their score compared to the normative distribution. This bar chart shows the mean and deviation of calculated T axis scores, arranged along the low to high spectrum of the axis. The do demonstrate ordinal increase along the low to high range.

In Figure 1, calculated axes scores are plotted for each axis across normative categories of the A/ASP to illustrate the non-ordinal nature of the BR axis and the ordinal nature of the T axis.

### 3.4. Comparison with External Measures

**Figure 2:**
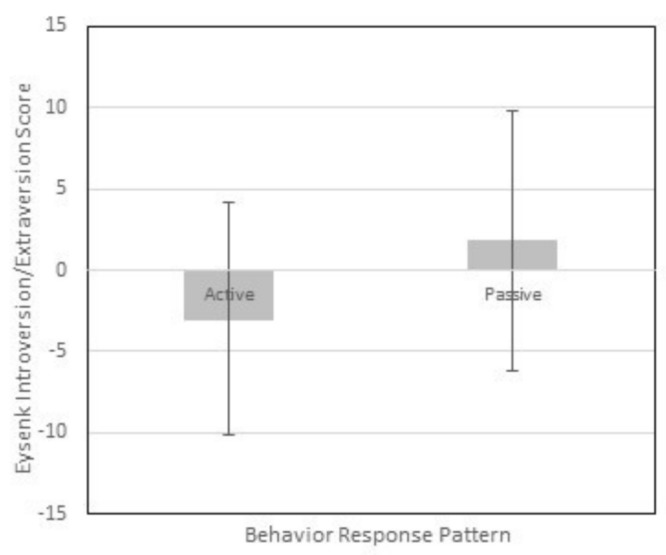
Introversion/Extraversion scores of participants with active and passive BR patterns. Mean and standard deviation of the Introversion/Extraversion axis scores of the EBQ-BV for participants categorized as having active or passive behavior response patterns according to the quadrant with the highest raw score on the A/ASP.

We explored whether variation along the model’s axes is consistent with variation in external measures of characteristics that may also reflect sensory processing as described by Dunn. We compared variation in behavioral response and threshold with variation in introversion/extraversion and neuroticism of axes of Eysenck’s personality model (Sato, 2005), respectively. As the behavioral response calculated axis scores did not vary according to participants’ classification under the A/ASP we formulated the hypothesis as: Participants with Active behavioral response patterns, as indicated by their maximum raw score, will have higher scores on the introversion/extraversion scale of the Eysenck Personality Questionnaire, Brief Version than participants with Passive behavioral response patterns. Although Introversion/Extraversion scores for participants with Passive behavioral response patterns were lower than those for participants with Active behavioral response patterns, the difference was not statistically significant; −3.0 ± 7.2 (*n* = 7) vs. 1.8 ± 8.0 (*n* = 131), *t*(136) = −1.554, *p* = 0.122, *r* = 0.13 (See Figure 2). This suggests that Eysenck’s concept of introversion/extraversion is distinct from Dunn’s behavioral response construct, despite being described in similar terms. However, as the distribution of scores yielded few participants in the passive category and the variability among them was high, this finding may also reflect a Type II error.

**Figure 3:**
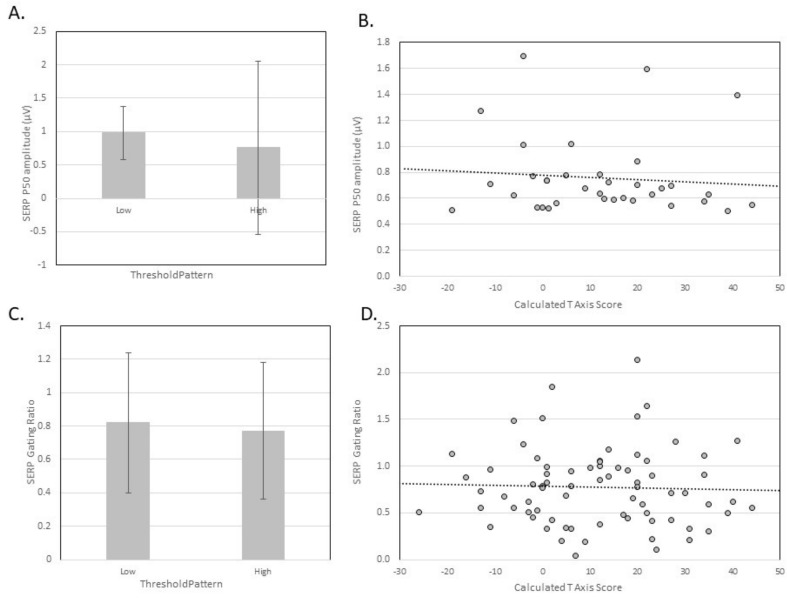
Relationship of somatosenory evoked potentials and gating to Dunn’s Threshold axis. (**A**) Mean and standard deviation of the SERP P50 amplitude for participants categorized as having low or high threshold patterns according to the quadrant with the highest raw score on the A/ASP. (**B**) Scatterplot of SERP P50 amplitude and calculated T axis scores. (**C**) Mean and standard deviation of the SERP gating ratio for participants categorized as having low or high threshold patterns according to the quadrant with the highest raw score on the A/ASP. (**D**) Scatterplot of SERP gating ratio and calculated T axis scores.

Somatosensory event related potentials (SERP) evoked by median nerve stimulation (MNS) were collected from 138 participants. A total of 38 participants had an average peak wave amplitude of at least 0.5 µV for the P50 SERP waveform when 200 MNS were presented in a train at 5 Hz. When these participants were categorized by highest raw score on the A/ASP, the amplitude of the averaged SERP P50 for participants with low threshold patterns was 0.98 ± 0.4 µV (*n* = 3) and for participants with High Threshold patterns 0.76 ± 1.3 µV (*n* = 35). This difference was not statistically significant (*t*(33) = −0.433, *p* = 0.668, *r* = 0.08). The calculated threshold and the amplitude of the P50 nonsignificant weak negative correlation (R = −0.089, *r^2^*= 0.007, *p* = 0.611). This suggests that the somatosensory evoked potential P50 was not a neurophysiological correlate of the threshold construct of the four-quadrant model of sensory processing in our sample. When MNS stimuli were presented in pairs, 72 participants had an average peak wave amplitude for the P50 of at least 0.5 µV for the first MNS stimulus in the pair. When participants were categorized by highest raw score on the A/ASP, the gating ratio for participants with Low threshold patterns was 0.82 ± 0.42 (*n* = 7) and for participants with High threshold patterns was 0.77 ± 0.41 (*n* = 67). This difference was not statistically significant (*t*(72) = 0.335, *p* = 0.738, *r* = 0.04). However, given the small sample of participants in the low threshold category, this may reflect a Type II error. The calculated threshold and the P50 gating ratio had nonsignificant weak negative correlation (R = −0.036, *r^2^*= 0.001, *p* = 0.764, *n* = 74). This suggests that somatosensory gating may not be a neurophysiological correlate of the threshold construct of the four-quadrant model of sensory processing. See Figure 3 for graphical depiction of these findings.

## 4. Discussion

Dunn’s four-quadrant model of sensory processing has clinical utility [2,4,5,16,17,18,19,20], and the associated standardized assessment has strong psychometrics. Here we examined the axis constructs that underlie the model, behavioral response and threshold, assessing them for scaling and relation to other traits. The findings suggest that behavioral response axis of Dunn’s model may not reflect a construct with ordinal range, suggesting that behavior response patterns may not reflect a spectrum of characteristic styles. Quadrant scores may reflect individuals’ most common response strategies, but across settings, they may use varieties of strategies, creating blur in scores on the axis. This may empower clinicians to take a top-down approach of teaching clients coping strategies that will help them deal with any sensory environment without beginning with bottom-up intervention strategies that change endogenous behavior response patterns.

Though passive and active behavior response patterns are described with language similar to Eysenck’s concepts of introversion and extraversion [24], there was no difference in I/E scores for participants with passive and active BR patterns. This suggests the concepts are distinct from one another; that Dunn’s model of may distinctly describe individuals’ behavior responses to sensory stimuli transmitted through the nervous system’s afferent systems, irrespective of their style of social engagement Although individuals assign meaning to sensory stimuli according to past experiences and current context [31], their behavior response may reflect momentary intention to tolerate, reduce, or intensify sensory stimulation. Further research is needed to explore the differences and similarities between behavior regulation and introversion/extraversion.

The threshold axis of Dunn’s model may reflect an ordinal range, with calculated threshold scores reflecting sensory processing patterns on a spectrum from low to high. This may suggest that threshold is a personal, endogenous characteristic. This may lend support for clinical interventions for individuals who are challenged by having a low or high threshold to employ bottom-up methods such as sensory integration therapy [32] where changes in threshold may result and translate into improved function.

In our findings, neither the amplitude of somatosensory evoked event related potentials and nor their gating differed by participants categorization using sensory profile scores or calculated threshold scores. This may suggest that in healthy, typical adults, the brain’s reaction to activation of a somatosensory pathway is not a direct correlate of Dunn’s concept of threshold. It may be that somatosensory processing in well-developed, healthy central nervous systems does not have much inter-individual variation that would create a range of SERP amplitude and gating. Our findings do not, however, rule out the use of EEG to detect immature or problematic sensory processing. Davies and colleagues (Davies & Gavin, 2007; Davies, Chang, & Gavin, 2010; Davies, Change, Gavin, 2009) demonstrated differences in sensory gating in children with clinically documented sensory processing disorders. Further research is needed to establish biomarkers and describe neurological underpinnings of sensory processing.

This study lends support to Dunn’s four-quadrant model as a conception of sensory processing patterns Though the behavior response axis did not show ordinal scaling, inspection of Figure 1A reveals that axis scores for participants passive response patterns were negative and those for participants with active response patterns were positive, suggesting distinctions between them. The threshold axis did present as a scale ranging from low to high. When calculated axes scores were plotted, the derived quadrant had high consistency with the quadrant in which participants had their highest raw score. Together, this may support the idea that individuals have a characteristic sensory processing pattern arising from the interaction of behavior response and threshold. This may support development of quadrant-specific interventions.

### Limitations

Participants represent a convenience sample. We recruited in the Midwest on a college campus and public/community locations via flyers and word of mouth. Flyers included a photograph of an individual wearing the EEG cap and referenced “sensory processing”. Individuals who chose to respond to recruiting may represent a different segment of society. We had few participants who were categorized as having either passive behavior regulation or low threshold patterns of processing. Perhaps individuals who typically employ passive behavior regulation are less likely to initiate research participation. It is also possible that individuals with low threshold judged the research procedures to be aversive. These possibilities may account for underrepresentation of these patterns in our sample. Conversely, these patterns may be uncommon in healthy, typical adults. In any case, not having a full range of response patterns may limit our ability to observe differences and trends in our data. Future research should include participants with broader ranges of sensory processing patterns. This may require research procedures to be modified to lower barriers to enrollment and enhance participant comfort. Researchers may want to specifically advertise their studies toward individuals who consider themselves to have passive behavior response and/or low threshold processing patterns. Inclusion of clinical populations, such as individuals with affective disorders or those with diagnosed sensory processing disorders, may extend the range of scores and allow patterns and trends to emerge from the analyses conducted here. In order to detect a large effect size, the one-way ANOVA needs an estimated sample size of 112 [33], which we had for the active BR and high T categories but fell far short of for the passive BR and low T categories.

Out of our sample of 138 participants for whom we had EEG data, only 38 and 74 had P50 waves of at least 0.5 µV for analysis of baseline and gating responses, respectively. This likely reflects use of a low intensity MNS. The MNS was well below the motor threshold in order to maximize participant comfort and compliance, as well as make the stimulus more similar to tactile stimulation rather than proprioceptive input, painful stimulus, or an electrical impulse in the median nerve. However, the intensity of MNS has been suggested to not have an influence on gating [34]. Still, in future studies, researchers may wish to use a more robust MNS stimulus or event related potentials evoked via other sensory modalities.

These limitations may have led to Type 2 errors, perhaps reflecting poor power to detect differences that would support they hypotheses. Therefore, we do not feel that the non-significant findings are not sufficient grounds for dismissing the axes constructs of Dunn’s model.

## Figures and Tables

**Table 1:** Participant demographics.

Characteristic	Sample (*n* = 139)
Age (years)	Average 27.19.4, range = 18–59
Gender	38 Male, 101 Female
Hand Dominance	122 Right, 17 Left
Reported Medications	82 none, 30 birth control, 13 psychiatric medications, 7 medications for hypertension, 6 medications for thyroid conditions, 5 medications for allergy, 4 medications for diabetes, 4 medications for digestive function, 3 medications for high cholesterol, 3 medications for respiratory function, 2 medications for kidney function, 2 nonsteroidal anti-inflammatory medications, 1 hormone treatment, 1 medication for migraine, 1 medication for sleep, 1 antibiotic

**Table 2:** Average calculated behavioral response axis scores for participants in each normative category of the Adult/Adolescent Sensory Profile.

Behavioral Response	Less Than Others	Similar to Others	More Than Others	Much More Than Others	ANOVA
Passive	(*n* = 0)	(*n* = 0)	−7.7 ± 9.2 (*n* = 3)	−9.0 ± 6.7 (*n* = 4)	F(1,5) = 0.05, ω = 0.40, *p* = 0.832
Active	13.7 ± 7.0 (*n* = 3)	18.2 ± 9.6 (*n* = 95)	20.5 ± 8.6 (*n* = 21)	17.8 ± 13.5 (*n* = 12)	F(3,127) = 0.572, ω = 0.10, *p* =0.634

**Table 3:** Average calculated threshold axis scores for participants in each normative category of the Adult/Adolescent Sensory Profile.

Threshold	Less Than Others	Similar to Others	More Than Others	Much More Than Others	ANOVA
Low	(*n* = 0)	−2.0 ± 2.8 (*n* = 2)	−8.3 ± 8.7 (*n* = 6)	−18.2 ± 13.1 (*n* = 6)	F(2,11) = 2.246, *p* = 0.152, ω = 0.39
High	11.0 ± 2.0 (*n* = 3)	11.9 ± 12.1 (*n* = 93)	18.5 ± 13.1 (*n* = 18)	21.6 + 20.2 (*n* = 10)	F(3,120) = 4.198, *p* = 0.007, ω = 0.27
